# (*E*)-2-[(5-Bromo-2-hydroxy­benzyl­idene)amino]benzonitrile

**DOI:** 10.1107/S1600536809027950

**Published:** 2009-07-22

**Authors:** Jian-Cheng Zhou, Nai-Xu Li, Chuan-Ming Zhang, Zheng-Yun Zhang

**Affiliations:** a College of Chemistry and Chemical Engineering, Southeast University, Nanjing 211189, People’s Republic of China

## Abstract

In the mol­ecule of the title compound, C_14_H_9_BrN_2_O, the dihedral angle between the aromatic rings is 1.09 (4)°. Intra­molecular O—H⋯N hydrogen bonding results in the formation of a planar (r.m.s. deviation = 0.0140 Å) six-membered ring. In the crystal structure, inter­molecular C—H⋯N inter­actions link the mol­ecules into chains.

## Related literature

For general background to Schiff base compounds in coordination chemistry, see: Chen *et al.* (2008 ▶); May *et al.* (2004 ▶); Weber *et al.* (2007 ▶). For a related structure, see: Elmalı *et al.* (1999 ▶). For bond-length data, see: Allen *et al.* (1987 ▶).
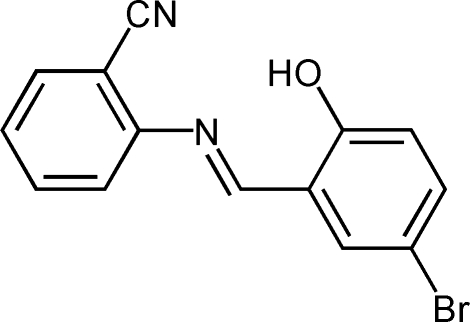


## Experimental

### 

#### Crystal data

C_14_H_9_BrN_2_O
*M* *_r_* = 301.14
Orthorhombic, 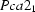

*a* = 25.609 (8) Å
*b* = 3.9299 (12) Å
*c* = 12.368 (4) Å
*V* = 1244.7 (7) Å^3^
*Z* = 4
Mo *K*α radiation
μ = 3.29 mm^−1^
*T* = 294 K
0.2 × 0.2 × 0.2 mm

#### Data collection

Bruker SMART CCD area-detector diffractometer
Absorption correction: multi-scan (*SADABS*; Bruker, 2000 ▶) *T*_min_ = 0.518, *T*_max_ = 0.518
9720 measured reflections
2771 independent reflections
1737 reflections with *I* > 2σ(*I*)
*R*_int_ = 0.048

#### Refinement

*R*[*F* ^2^ > 2σ(*F* ^2^)] = 0.039
*wR*(*F* ^2^) = 0.090
*S* = 1.01
2771 reflections
163 parameters
1 restraint
H-atom parameters constrained
Δρ_max_ = 0.23 e Å^−3^
Δρ_min_ = −0.29 e Å^−3^
Absolute structure: Flack (1983 ▶), 1271 Friedel pairs
Flack parameter: 0.039 (14)

### 

Data collection: *SMART* (Bruker, 2000 ▶); cell refinement: *SAINT* (Bruker, 2000 ▶); data reduction: *SAINT*; program(s) used to solve structure: *SHELXS97* (Sheldrick, 2008 ▶); program(s) used to refine structure: *SHELXL97* (Sheldrick, 2008 ▶); molecular graphics: *ORTEP-3 for Windows* (Farrugia, 1997 ▶) and *PLATON* (Spek, 2009 ▶); software used to prepare material for publication: *SHELXL97*.

## Supplementary Material

Crystal structure: contains datablocks I, global. DOI: 10.1107/S1600536809027950/hk2739sup1.cif

Structure factors: contains datablocks I. DOI: 10.1107/S1600536809027950/hk2739Isup2.hkl

Additional supplementary materials:  crystallographic information; 3D view; checkCIF report

## Figures and Tables

**Table 1:** Hydrogen-bond geometry (Å, °)

*D*—H⋯*A*	*D*—H	H⋯*A*	*D*⋯*A*	*D*—H⋯*A*
O1—H1*A*⋯N1	0.82	1.93	2.651 (4)	146
C7—H7*A*⋯N2^i^	0.93	2.44	3.326 (4)	160

Symmetry code: (i) 
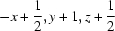
.

**Fig. 1.**
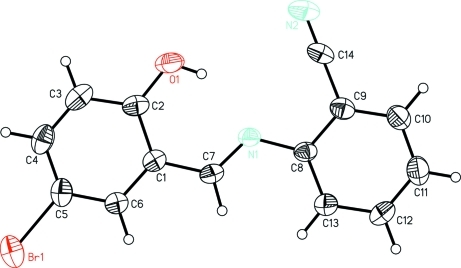
The molecular structure of the title molecule, with the atom-numbering scheme. Hydrogen bond is shown as dashed line.

**Fig. 2.**
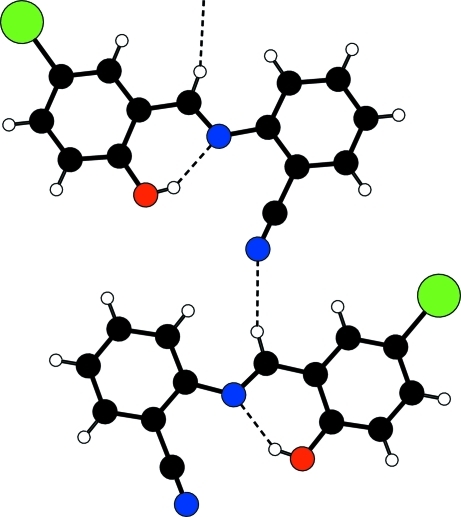
A partial packing diagram of the title compound. Hydrogen bonds are shown as dashed lines.

## supplementary crystallographic information

### Comment

Schiff base compounds have received considerable attention for many years, primarily due to their importance in the development of coordination chemistry related to magnetism (Weber *et al.*,  2007), catalysis (Chen *et al.*,  2008) and biological process (May *et al.*,  2004). We report herein the synthesis and crystal structure of the title compound.

In the molecule of the title compound (Fig. 1), the bond lengths (Allen *et al.*,  1987) and angles are within normal ranges and comparable with the corresponding values in a similar compound (Elmalı *et al.*, 1999). Rings A (C1-C6) and B (C8-C13) are, of course, planar, and they are oriented at a dihedral angle of A/B = 1.09 (4)°. Intramolecular O-H···N hydrogen bond (Table 1) results in the formation of planar six-membered ring C (O1/N1/C1/C2/C7/H1A), it is oriented with respect to rings A and B at dihedral angles of A/C = 2.00 (4) and B/C = 1.42 (4) °. So, rings A, B and C are almost coplanar.

In the crystal structure, intermolecular C-H···N interactions link the molecules into chains (Fig. 2), , in which they may be effective in the stabilization of the structure.

### Experimental

For the preparation of hte title compound, 2-aminobenzonitrile (0.472 g, 4 mmol) and 5-bromo-2-hydroxybenzaldehyde (0.8 g, 4 mmol) were dissolved in ethanol (20 ml). The mixture was heated to reflux for 5 h, and then cooled to room temperature. The solution was filtered and after two weeks yellow crystals suitable for X-ray analysis were obtained.

### Refinement

H atoms were positioned geometrically with O-H = 0.82 Å (for OH) and C-H = 0.93 Å for aromatic H atoms, respectively and constrained to ride on their parent atoms, with U_iso_(H) = xU_eq_(C,O), where x = 1.5 for OH H and x = 1.2 for aromatic H atoms.

### Crystal data

**Table:**

C_14_H_9_BrN_2_O	*F*(000) = 600
*M**_r_* = 301.14	*D*_x_ = 1.607 Mg m^−^^3^
Orthorhombic, *P**c**a*2_1_	Mo *K*α radiation, λ = 0.71073 Å
Hall symbol: P 2c -2ac	Cell parameters from 1688 reflections
*a* = 25.609 (8) Å	θ = 3.1–27.7°
*b* = 3.9299 (12) Å	µ = 3.29 mm^−^^1^
*c* = 12.368 (4) Å	*T* = 294 K
*V* = 1244.7 (7) Å^3^	Prism, yellow
*Z* = 4	0.2 × 0.2 × 0.2 mm

### Data collection

**Table:**

Bruker SMART CCD area-detector diffractometer	2771 independent reflections
Radiation source: fine-focus sealed tube	1737 reflections with *I* > 2σ(*I*)
graphite	*R*_int_ = 0.048
Detector resolution: 13.6612 pixels mm^-1^	θ_max_ = 27.6°, θ_min_ = 1.6°
φ and ω scans	*h* = −33→33
Absorption correction: multi-scan (*SADABS*; Bruker, 2000)	*k* = −5→5
*T*_min_ = 0.518, *T*_max_ = 0.518	*l* = −16→14
9720 measured reflections	

### Refinement

**Table:**

Refinement on *F*^2^	Secondary atom site location: difference Fourier map
Least-squares matrix: full	Hydrogen site location: inferred from neighbouring sites
*R*[*F*^2^ > 2σ(*F*^2^)] = 0.039	H-atom parameters constrained
*wR*(*F*^2^) = 0.090	*w* = 1/[σ^2^(*F*_o_^2^) + (0.0283*P*)^2^ + 0.0106*P*] where *P* = (*F*_o_^2^ + 2*F*_c_^2^)/3
*S* = 1.01	(Δ/σ)_max_ < 0.001
2771 reflections	Δρ_max_ = 0.23 e Å^−^^3^
163 parameters	Δρ_min_ = −0.29 e Å^−^^3^
1 restraint	Absolute structure: Flack (1983), 1271 Friedel pairs
Primary atom site location: structure-invariant direct methods	Flack parameter: 0.039 (14)

### Special details

**Table:**

Geometry. All e.s.d.'s (except the e.s.d. in the dihedral angle between two l.s. planes) are estimated using the full covariance matrix. The cell e.s.d.'s are taken into account individually in the estimation of e.s.d.'s in distances, angles and torsion angles; correlations between e.s.d.'s in cell parameters are only used when they are defined by crystal symmetry. An approximate (isotropic) treatment of cell e.s.d.'s is used for estimating e.s.d.'s involving l.s. planes.
Refinement. Refinement of *F*^2^ against ALL reflections. The weighted *R*-factor *wR* and goodness of fit *S* are based on *F*^2^, conventional *R*-factors *R* are based on *F*, with *F* set to zero for negative *F*^2^. The threshold expression of *F*^2^ > σ(*F*^2^) is used only for calculating *R*-factors(gt) *etc*. and is not relevant to the choice of reflections for refinement. *R*-factors based on *F*^2^ are statistically about twice as large as those based on *F*, and *R*- factors based on ALL data will be even larger.

### Fractional atomic coordinates and isotropic or equivalent isotropic displacement parameters (Å^2^)

**Table:**

	*x*	*y*	*z*	*U*_iso_*/*U*_eq_	
Br1	0.014802 (15)	0.46800 (11)	0.49637 (9)	0.0816 (2)	
O1	0.15855 (14)	−0.0121 (8)	0.1439 (2)	0.0767 (9)	
H1A	0.1892	0.0184	0.1605	0.115*	
N1	0.23690 (11)	0.1858 (9)	0.2691 (3)	0.0471 (7)	
N2	0.28448 (17)	−0.1697 (11)	0.0417 (3)	0.0800 (12)	
C1	0.14763 (14)	0.2551 (10)	0.3176 (3)	0.0481 (9)	
C2	0.12781 (19)	0.1038 (11)	0.2221 (4)	0.0567 (12)	
C3	0.0737 (2)	0.0789 (11)	0.2121 (4)	0.0723 (13)	
H3A	0.0598	−0.0122	0.1490	0.087*	
C4	0.04062 (17)	0.1828 (11)	0.2912 (4)	0.0679 (12)	
H4A	0.0047	0.1582	0.2825	0.081*	
C5	0.06036 (14)	0.3244 (10)	0.3842 (4)	0.0576 (10)	
C6	0.11302 (15)	0.3625 (11)	0.3970 (3)	0.0543 (10)	
H6A	0.1259	0.4619	0.4598	0.065*	
C7	0.20246 (14)	0.2971 (10)	0.3346 (3)	0.0488 (9)	
H7A	0.2135	0.4110	0.3964	0.059*	
C8	0.29066 (13)	0.2276 (9)	0.2900 (3)	0.0458 (9)	
C9	0.32433 (17)	0.1123 (10)	0.2105 (3)	0.0523 (10)	
C10	0.37810 (18)	0.1385 (12)	0.2213 (4)	0.0622 (12)	
H10A	0.4001	0.0623	0.1666	0.075*	
C11	0.39839 (16)	0.2791 (13)	0.3143 (4)	0.0735 (13)	
H11A	0.4344	0.2974	0.3227	0.088*	
C12	0.36610 (17)	0.3907 (12)	0.3933 (4)	0.0661 (12)	
H12A	0.3802	0.4864	0.4556	0.079*	
C13	0.31227 (16)	0.3646 (12)	0.3830 (4)	0.0613 (11)	
H13A	0.2907	0.4392	0.4386	0.074*	
C14	0.30179 (18)	−0.0458 (12)	0.1155 (4)	0.0598 (11)	

### Atomic displacement parameters (Å^2^)

**Table:**

	*U*^11^	*U*^22^	*U*^33^	*U*^12^	*U*^13^	*U*^23^
Br1	0.0607 (2)	0.0846 (3)	0.0995 (4)	0.0101 (2)	0.0174 (3)	0.0112 (4)
O1	0.086 (2)	0.096 (3)	0.0480 (19)	−0.0127 (17)	−0.0052 (17)	−0.0184 (16)
N1	0.0530 (19)	0.054 (2)	0.0344 (18)	−0.0040 (14)	0.0005 (15)	−0.0044 (16)
N2	0.106 (3)	0.081 (3)	0.054 (3)	0.002 (2)	0.007 (2)	−0.024 (2)
C1	0.055 (2)	0.044 (2)	0.045 (2)	−0.0054 (19)	−0.005 (2)	0.0047 (19)
C2	0.073 (3)	0.050 (3)	0.047 (3)	−0.009 (2)	−0.004 (3)	−0.005 (2)
C3	0.075 (3)	0.076 (3)	0.067 (3)	−0.015 (3)	−0.024 (3)	0.004 (3)
C4	0.052 (2)	0.060 (3)	0.091 (4)	−0.010 (2)	−0.015 (3)	0.009 (3)
C5	0.049 (2)	0.048 (2)	0.076 (3)	0.0014 (18)	−0.001 (2)	0.016 (2)
C6	0.056 (2)	0.056 (3)	0.052 (3)	−0.0010 (19)	−0.003 (2)	−0.006 (2)
C7	0.059 (2)	0.049 (2)	0.038 (2)	−0.0061 (18)	−0.006 (2)	−0.002 (2)
C8	0.057 (2)	0.042 (2)	0.038 (2)	−0.0070 (17)	0.000 (2)	0.0003 (19)
C9	0.066 (3)	0.046 (2)	0.045 (3)	0.0001 (19)	0.005 (2)	0.004 (2)
C10	0.062 (3)	0.061 (3)	0.064 (3)	0.000 (2)	0.012 (3)	−0.003 (3)
C11	0.054 (2)	0.077 (3)	0.089 (4)	−0.003 (2)	0.010 (3)	0.000 (3)
C12	0.065 (3)	0.069 (3)	0.064 (3)	−0.009 (2)	−0.018 (2)	−0.008 (2)
C13	0.057 (2)	0.076 (3)	0.051 (3)	−0.006 (2)	0.001 (2)	−0.009 (2)
C14	0.076 (3)	0.059 (3)	0.045 (3)	0.005 (2)	0.014 (2)	−0.007 (2)

### Geometric parameters (Å, °)

**Table:**

Br1—C5	1.898 (4)	C7—C1	1.429 (5)
O1—C2	1.328 (6)	C7—H7A	0.9300
O1—H1A	0.8200	C8—C9	1.385 (5)
N1—C7	1.275 (4)	C8—C13	1.384 (5)
N1—C8	1.411 (4)	C9—C10	1.387 (6)
C2—C1	1.416 (6)	C10—C11	1.377 (6)
C3—C2	1.393 (7)	C10—H10A	0.9300
C3—C4	1.358 (7)	C11—H11A	0.9300
C3—H3A	0.9300	C12—C11	1.354 (6)
C4—H4A	0.9300	C12—H12A	0.9300
C5—C4	1.374 (6)	C13—C12	1.388 (6)
C6—C1	1.388 (5)	C13—H13A	0.9300
C6—C5	1.366 (5)	C14—N2	1.125 (5)
C6—H6A	0.9300	C14—C9	1.449 (7)
			
C2—O1—H1A	109.5	N1—C7—H7A	118.5
C7—N1—C8	121.2 (3)	C1—C7—H7A	118.5
C2—C1—C7	121.6 (4)	C9—C8—N1	116.0 (3)
C6—C1—C2	119.2 (4)	C9—C8—C13	117.9 (3)
C6—C1—C7	119.2 (4)	C13—C8—N1	126.0 (3)
O1—C2—C1	122.6 (4)	C8—C9—C10	121.7 (4)
O1—C2—C3	120.0 (4)	C8—C9—C14	118.0 (4)
C3—C2—C1	117.4 (4)	C10—C9—C14	120.4 (4)
C2—C3—H3A	118.8	C9—C10—H10A	120.5
C4—C3—C2	122.4 (5)	C11—C10—C9	119.0 (4)
C4—C3—H3A	118.8	C11—C10—H10A	120.5
C3—C4—C5	119.7 (4)	C10—C11—H11A	119.9
C3—C4—H4A	120.2	C12—C11—C10	120.2 (4)
C5—C4—H4A	120.2	C12—C11—H11A	119.9
C4—C5—Br1	120.4 (3)	C11—C12—C13	121.0 (4)
C6—C5—Br1	119.3 (3)	C11—C12—H12A	119.5
C6—C5—C4	120.3 (4)	C13—C12—H12A	119.5
C1—C6—H6A	119.5	C8—C13—C12	120.2 (4)
C5—C6—C1	121.0 (4)	C8—C13—H13A	119.9
C5—C6—H6A	119.5	C12—C13—H13A	119.9
N1—C7—C1	123.1 (3)	N2—C14—C9	179.7 (5)

### Hydrogen-bond geometry (Å, °)

**Table:**

*D*—H···*A*	*D*—H	H···*A*	*D*···*A*	*D*—H···*A*
O1—H1A···N1	0.82	1.93	2.651 (4)	146
C7—H7A···N2^i^	0.93	2.44	3.326 (4)	160

Symmetry codes: (i) −*x*+1/2, *y*+1, *z*+1/2.
